# Targeting FGL2 in glioma immunosuppression and malignant progression

**DOI:** 10.3389/fonc.2022.1004700

**Published:** 2022-10-13

**Authors:** Xiaoyu Ma, Hongtao Zhu, Lidong Cheng, Xin Chen, Kai Shu, Suojun Zhang

**Affiliations:** ^1^ Department of Neurosurgery, Tongji Hospital, Tongji Medical College, Huazhong University of Science and Technology, Wuhan, China; ^2^ Department of Oncology, Tongji Hospital, Tongji Medical College, Huazhong University of Science and Technology, Wuhan, China

**Keywords:** FGL2, resident immune cell, glioma microenvironment, immunotherapy, immune checkpoint molecules

## Abstract

Glioblastoma (GBM) is the most malignant type of glioma with the worst prognosis. Traditional therapies (surgery combined with radiotherapy and chemotherapy) have limited therapeutic effects. As a novel therapy emerging in recent years, immunotherapy is increasingly used in glioblastoma (GBM), so we expect to discover more effective immune targets. FGL2, a member of the thrombospondin family, plays an essential role in regulating the activity of immune cells and tumor cells in GBM. Elucidating the role of FGL2 in GBM can help improve immunotherapy efficacy and design treatment protocols. This review discusses the immunosuppressive role of FGL2 in the GBM tumor microenvironment and its ability to promote malignant tumor progression while considering FGL2-targeted therapeutic strategies. Also, we summarize the molecular mechanisms of FGL2 expression on various immune cell types and discuss the possibility of FGL2 and its related mechanisms as new GBM immunotherapy.

## Introduction

Gliomas are the most universal malignant central nervous system (CNS) tumors, accounting for approximately 80% of all brain malignancies (1, 2). According to the classification of central nervous system tumors in the 2021 World Health Organization (WHO), Adult-type diffuse gliomas are classified into three types: Astrocytoma, IDH-mutant; Oligodendroglioma, IDH-mutant, and 1p/19q-co deleted; Glioblastoma, IDH-wildtype (3, 4). Among these, glioblastoma (GBM) is one of the most lethal gliomas, accounting for 70% of all widespread glioma diagnoses, with a median survival time of 15 months (5). Currently, conventional treatments for gliomas include surgery, chemotherapy, and radiotherapy. However, the 5-year survival rate of GBM patients is rarely 6.8% due to the infiltrative growth, aggressiveness, and recurrence of the malignant gliomas (6–8). The extremely low survival rate of GBM patients has prompted a search for more effective drugs and treatments (9).

Immunotherapy is an innovative treatment method in tumors today (10). The innate and adaptive immune system in the host recognizes and kills tumor cells in the initial stage of tumorigenesis, some tumor cells survive into the equilibrium phase, and in the later stages, tumor cells become resistant to the body’s immune response and immune cells escape. It is based on the study of tumor immune cell escape mechanisms that immunotherapy has developed. Immunotherapy attempts to activate immune cells by reactivating the tumor immune system and blocking immune escape pathways (11, 12).Targeted cytokines, immune checkpoint inhibitors (ICIs), pericyte therapy, oncolytic viruses, and cancer vaccines are currently used in clinical treatment and have been verified to be helpful in fighting tumor cells (13). Due to the success of immunotherapy in melanoma and hematologic tumors, attempts have been made to understand GBM in terms of the immune microenvironment and immune response and to improve its prognosis (14).Unfortunately, CTLA-4 monoclonal antibody Ipilimumab, PD-1 monoclonal antibody Nivolumab and so on have shown in clinical trials limited efficacy against GBM (15).

Due to the complex immune microenvironment of glioma, immunotherapy of glioma has been increasingly studied in recent years, and some promising results have been achieved. (16). In brain tumors, tumor cells secrete a large number of cytokines, growth factors and chemokines that promote the entry of numerous non-tumor cells such as infiltrating immune cells, pericytes, astrocytes, oligodendrocytes, and endothelial cells into the tumor (16–18). These cells and the various factors constitute the tumor microenvironment, which plays a crucial role in promoting tumor development, metastasis, and resistance to cancer therapy. The complex immune microenvironment of glioma makes it an excellent challenge for immunotherapy (19). In order to evade immune surveillance and clearance, tumor cells can suppress anti-tumor immunity through cancer immune editing, by which the immune response shifts from preventing the development of cancer to promoting the growth of tumor cells, thus evading immune surveillance (20, 21).

FGL2 is an important pleiotropic immunomodulatory cytokine discovered in recent years, which in cancer achieves immunosuppression by inhibiting antigen-presenting cells (APCs), suppressing T cell proliferation, inducing macrophage polarization to M2 and inducing regulatory T cell (TREG) activity (22, 23). However, the role of FGL2 in TME and the therapeutic potential of targeting this cytokine in gliomas is unclear. Here, we review the role of FGL2 in brain cancer and discuss its role in immunosuppression and tumor progression. We examined the molecular mechanisms responsible for regulating FGL2 expression in various cell types and considered the possibility that immunotherapies developed against this cytokine may improve the prognosis of GBM patients.

## Biogenesis of FGL2

Fibrinogen-like protein 2 (Fgl2), as a number of the fibrinogen-associated protein superfamily, has a molecular weight of approximately 64 KD (24). It was first identified in the 1990s and was initially thought to be secreted by a constitutively expressed cytotoxic T lymphocyte. The protein encoded by FGL2 is homologous to the β and γ chains of fibrinogen at its carboxy terminus and carboxy terminus, with a FRED structure (25). Numerous studies surrounding FGL2 have revealed many functions of it. For example, Th1 and Th2 cytokines trigger the coagulation system by acting on FGL2 on endothelial cells, FGL2 can directly activate prothrombin to produce thrombin. However, FGL2, which is secreted by peripheral blood CD4+ and CD8+ T cells, has no coagulation activity (26). It can inhibit the maturation of dendritic cells and the proliferation of T cells and has immunosuppressive activity. Due to the two activities exhibited by FGL2, it is now more uniform to categorize FGL2 as a membrane-bound protein and a secretory protein, with the membrane-bound type have coagulation activity, and the secretory type with immunomodulatory function (27, 28). Previous studies have shown that FGL2 plays an important role in inflammatory diseases such as severe viral hepatitis, rheumatoid arthritis, chronic obstructive pulmonary disease (COPD) (28),and inflammatory bowel disease (IBD) (29). At the same time, FGL2 is implicated in the malignant progression of tumors in hepatocellular carcinoma, central nervous system tumors (22), breast cancer, ovarian cancer and so on.

Recent studies have shown that FGL2 can contribute to the growth of gliomas by inducing multiple immune mechanisms (30). However, the role of FGL2 in gliomas and the therapeutic potential to target this protein in cancer patients remains unclear. Therefore, this paper reviews the role of FGL2 in glioma and discusses its role in immunosuppression and malignant progression. We summarize the molecular mechanisms by which FGL2 may be expressed in various cell types and explore the potential prognostic value and immunotherapeutic targeting of FGL2 in gliomas.

## FGL2 and glioma

**Figure 1:**
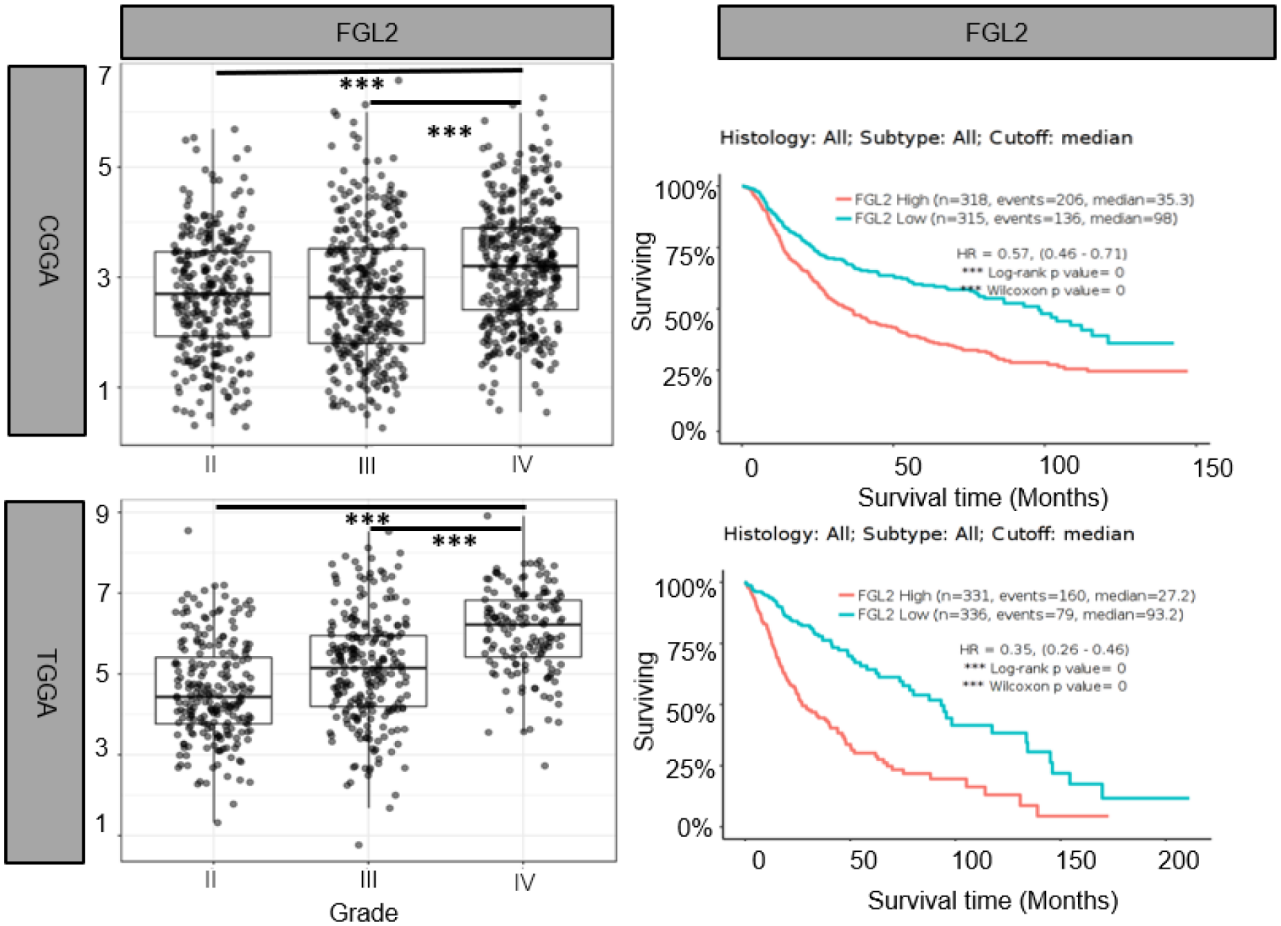
Elevated FGL2 mRNA levels in samples from patients with glioma and associated with poor patient prognosis. Analysis of glioma patient samples from The Chinese Glioma Genome Atlas (CCGA) and The Cancer Genome Atlas (TCGA) cohorts. FGL2 mRNA expression levels increased with glioma grade (II, III, IV). The figure was excerpted from GlioVis (http://gliovis.bioinfo.cnio.es/).

Yan et al. were the first to identify the strong expression of FGL2 in gliomas and conducted experiments to prove it (30). They found that 83.8% of GBM patients had FGL2 gene amplification or copy increase, and 72.5% of LGG patients had diploidy of the FGL2 gene. They divided GBM patients into FGL2 High and FGL2 Low groups according to FGL2 mRNA expression levels. The results showed that the overall survival rate of patients in the FGL2 High group was lower than that of patients in the FGL2 Low group. The median survival times for low and high expression of FGL2 were 394 and 357 days. The 5-year survival rate was estimated to be 4.98% for patients with low FGL2 expression and 0.99% for patients with high FGL2 expression. Song et al. found by immunohistochemistry that the expression of FGL2 was significantly higher in glioma tissues than in normal tissues (31). And we also found that FGL2 mRNA expression levels were higher in high-grade gliomas than in low-grade gliomas, and low expression of FGL2 increased patient survival and prolonged patient survival time based on the Chinese Glioma Genome Atlas (CGGA) (http://www.cgga.org.cn/) and the Cancer Genome Atlas (TCGA)(http://cancergenome.nih.gov/) databases.(**Figure 1**) These data suggest that FGL2 expression in glioma is positively correlated with tumor malignancy and patient survival.

Although the cellular origin of FGL2 in gliomas has not been determined, it has been shown that FGL2 is higher expressed in glioblastoma and glioblastoma stem cells (GSCs) compared to fibroblast cells. In contrast, there is no significant difference in FGL2 expression between glioblastoma and GSC line (22, 31).Using murine glioma models, Yan et al. verified that FGL2 might promote glioma growth in murine models by inhibiting the infiltration of immunosuppressive cells in the tumor microenvironment and that neutralization of FGL2 protein using anti-FGL2 antibody prolonged the survival time of mic (30). FGL2 secreted by GSCs is thought to activate tumor-infiltrating immune cells. For example, Fgl2 can immunomodulate the organism by inhibiting T cell differentiation and proliferation, DCs maturation, CD8+ T cell function, complement activation and promoting B cell apoptosis, etc. (22, 23, 30, 31). In addition, FGL2 overexpression can skew macrophages and other activated antigen-presenting cells (APCs) in the tumor microenvironment from an inflammatory (M 1) or neoplastic (M0) phenotype to an immunosuppressive (M2) phenotype (23). The limitation of the current study is that the exact mechanism of FGL2-induced immunity is unclear and needs further investigation.

## FGL2 and tumor progression

As well as its immunomodulatory functions of immunosuppressive and immunostimulatory activities, FGL2 can promote tumor progression by increasing cancer cell proliferation (32).In the mouse model of lung cancer, FGL2 promotes tumor growth by stimulating angiogenesis, immunosuppression, and tumor cell proliferation (33).Similarly, *in vitro* studies showed a dose-dependent increase in FGL2-mediated cell proliferation in LOVO and SW620 colorectal cancer cells, and knockdown of FGL2 resulted in reduced proliferation, migration and invasion (34, 35).Overexpression of FGL2 can eliminate the decrease in cell proliferation, migration, and aggression caused by the MAPK signaling inhibitor U0126 (34).In hepatocellular carcinoma studies, investigators used recombinant hFGL2 protein to stimulate HCCLM6 cells with thrombospondin and Ca2+, then found that phosphorylated p38 and ERK were significantly upregulated, while this upregulation could be abrogated by hirudin (36). Stable downregulation of FGL2 expression in the FGL2 knockdown HCCLM6 cell line was found to result in delayed tumor growth and reduced angiogenesis, along with decreased VEGF I and L-8 expression. These findings suggest that FGL2 may regulate HCCLM6 tumor cell growth by affecting angiogenesis (36). However, this phenomenon was not reproduced *in vivo*.

## Interaction of FGL2 with resident immune cells in glioma

**Figure 2:**
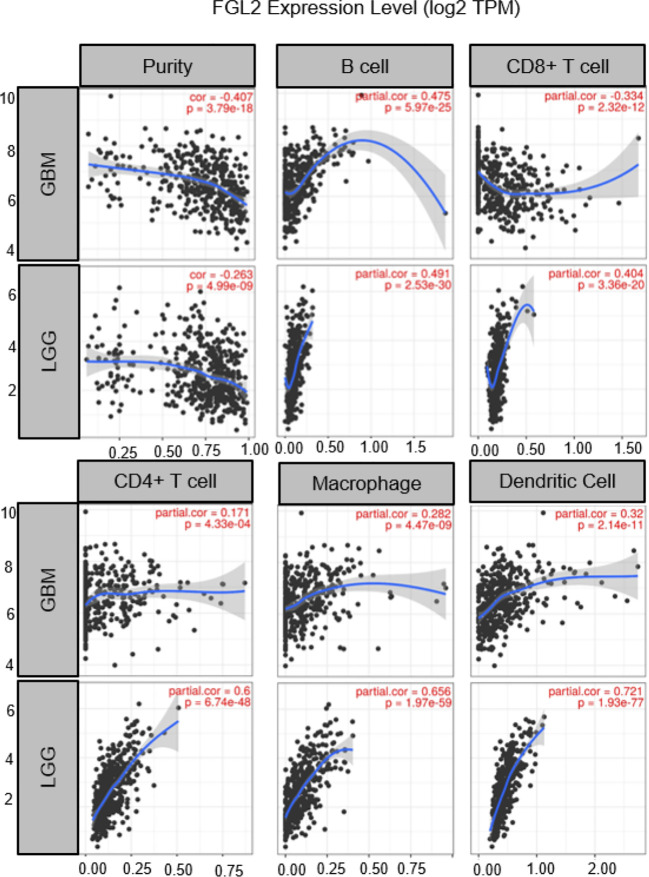
Correlation of FGL2 with immune cells. The figure was excerpted from TIMER (https://cistrome.shinyapps.io/timer/).

**Figure 3:**
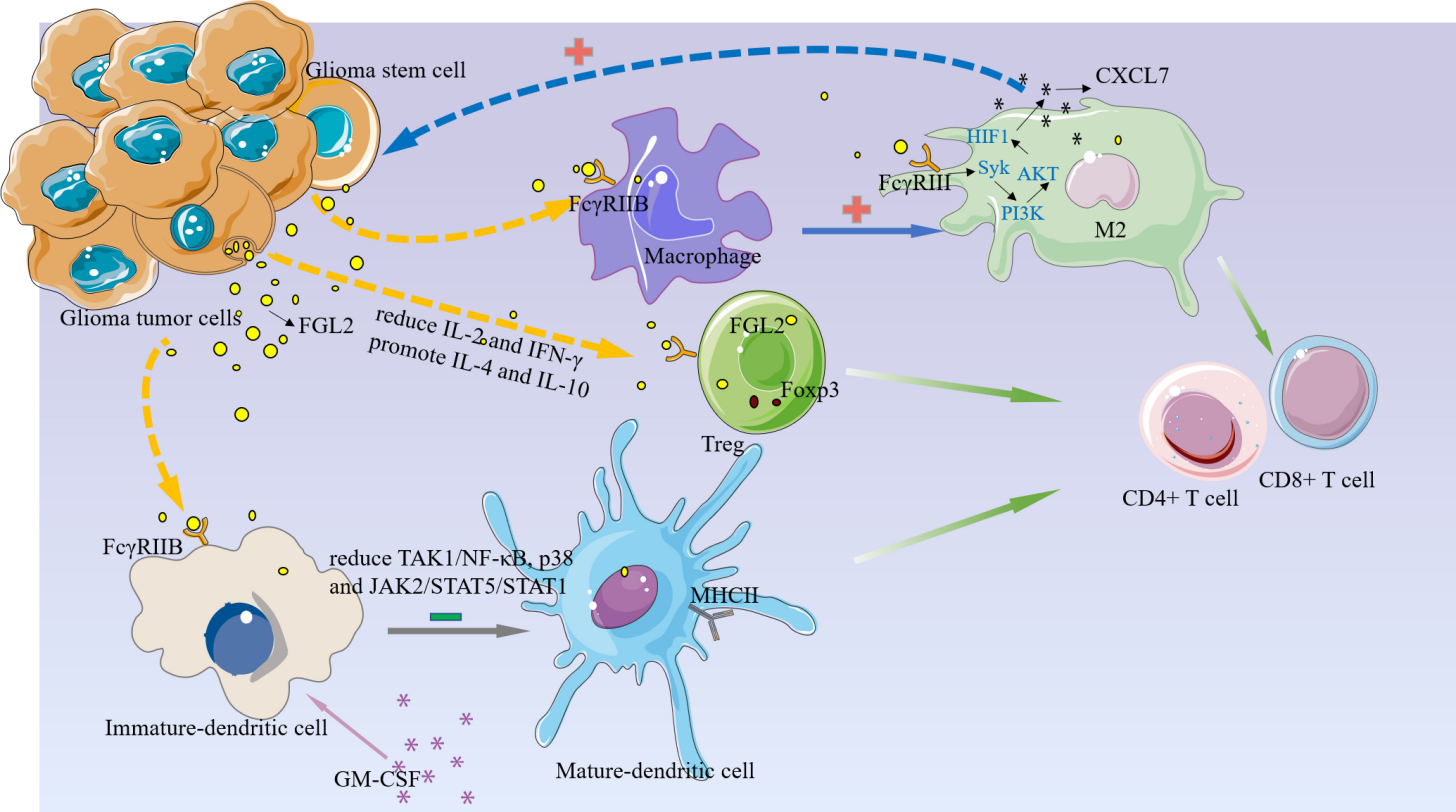
FGL2 plays an immunosuppressive function in the glioma microenvironment.1, FGL2 secreted by tumor cells binds to FCGR2B on immature DC cells and inhibits DC cell maturation and thus T cell proliferation by blocking the TAK1/NF-κB, p38, and JAK2/STAT5/STAT1 pathways.2, FGL2 inhibits the immune activators IL-2 and IFN-γ production, promotes immunosuppressive factors IL-4 and IL-10, increases Treg, and inhibits T-cell activation.3, FGL2 induces macrophages toward the M2 phenotype and produces CXCL7 in M2 cells *via* the FCGR2B/Syk/PI3K/AKT/HIF1α pathway, and CXCL7, in turn, acts on GSC to promote glioma cell stemness maintenance.

Although the origin of FGL2 in glioma is not fully understood, it has been shown to be expressed mainly in immune cells such as endothelial cells, macrophages, NK cells, T cells and tumor cells (27, 37). Low levels of FGL2 expression are associated with high granulocyte-macrophage (GM-CSF) colony-stimulating factor expression (38). Overexpression of FGL2 in a mouse glioma model was observed to increase CD4+FoxP3tregs cells and induced macrophages toward M2 phenotype shift. Ultimately, T cell initiation capacity was decreased (30).Yan et al. found that knockdown of FGL2 in immunoreactive mice reversed immune dysfunction in dendritic cells (DCs) and induced differentiation of CD103+ DCs in the brain (37). In addition, FGL2 has also been shown to play an important role in regulating myeloid-derived suppressor cells (MDSCs) (39, 40).FGL2 also was found to have a strong correlation with both immune cells in the TIMER database. (**Figure 2**) These findings suggest that FGL2 exerts its immunosuppressive effects through a variety of tumor-mediated immunosuppressive mechanisms (**Figure 3**).

### FGL2 inhibits DC maturation

Among the immune cells involved in the immune response, dendritic cells (DC) are the most functional antigen-presenting cells known (41–44). It can effectively extract, process, treat, and present antigens to T and B lymphocytes to activate the body’s specific immunity (45). DC presents a large number of tumor antigen peptides so that the corresponding T cell receptors are fully occupied; binds to T cells, facilitating T cell clearance of tumors; promotes T cell enrichment and enhances activation of T cells; fully activates T cells (46). Immature DCs can be released into tissues *via* blood vessels and are constantly searching for invading antigens and mutated cells. Once found, DCs immediately function to engulf these antigens and cells while digesting them with digestive enzymes from intracellular lysosomes. Digested foreign bodies and cells leave behind fragments of biomolecules that DCs will put on MHC molecules on the cell surface and enter the lymphoid tissue for T cell recognition (47–49).. In this process, DC cells are gradually transformed into mature antigen-presenting cells, a process often referred to as “antigen presentation”. And studies in recent years have shown that DC cells are closely linked to the prognosis of tumors (50). In solid tumors, patients with a high number of DC cell infiltrations tend to have a better prognosis (51).Therefore, DC cells are also used in research for tumor treatment, such as DC vaccines (52).

The most abundant and intrinsically stimulatory migrating cell type is the CD103+ DC population. In melanoma, CD103+ DCs transport tumor antigens to Draining lymph nodes (dLN) in a CCR7-dependent manner, resulting in increased numbers of tumor-specific CD8+ T cells (53). FGL2 promotes glioma progression by inhibiting CD103+ dendritic cell differentiation (22). In tumors, tumor cells release antigen to bind with antigen-presenting cells APCs, which further activate T cells, and activated CD8+ T lymphocytes exert the ability to recognize and kill tumor cells (54). APCs express major histocompatibility class II (MHCII) molecules, including DCs, macrophages, monocytes, B lymphocytes, and microglia in the brain (55). Batf3 is a key transcription factor that regulates CD103+/CD8a+ DC differentiation (56).The study by Quintana et al. shows that in the absence of Batf3, there is an impairment in the recognition of tumor cells by T cells. GM-CSF promotes the differentiation of myeloid cells and plays an important role in the differentiation, proliferation and activation of DCs (57). Yan Jun et al. found that GM-CSF induced TAK1/NF-κB, p38 and JAK2/STAT5/STAT1 signaling to induce CD103 expression. And a small amount of FGL2 expression in tumor tissues could significantly reduce the intensity of these CD103+DC-induced signals (22).This discovery may shed new light on the development of DC immunotherapy.

### FGL2 and tumor-associated T cell

T cells can shape immune responses in tumors, autoimmunity and infections, where CD4+ T (Th) cells and CD8+ T cells mediate effector responses that are suppressed by regulatory T (Treg) cells (58, 59). The balance between the effector T cell and Treg cell functions coordinates immune homeostasis and regulation of immune function. It has a vital role in tumorigenesis and development. Depending on the information the environment delivers, T cell metabolism dynamically changes and determines various aspects of its functional differentiation. CD4+ or CD8+ T cells recognize tumor antigens and autoantigens by expressing αβ T cell receptors (TCRs) and therefore play a crucial role in shaping the immune response in the formation of cancer or autoimmune diseases (60, 61). Following stimulation by cognate antigens, T cells are activated, proliferate, and undergo functional differentiation in response to environmental information. Initial CD8+ T cells without antigen stimulation differentiate into cytotoxic effector cells and long-term memory cells (62, 63). Initial CD4+ T cells differentiate into Th1, Th2, Th17, and Th effector cells, which can also form long-term memory cells, and immunosuppressive Treg cells expressing Foxp3 (64, 65). Cancer development is often associated with an immunosuppressive tumor microenvironment (TME), in which tumor-specific cytotoxic CD8+ T cells are often inadequate or dysfunctional and unable to eradicate malignant cells. T cell activation requires APCs such as CD103+ DCs to present antigens to T cells, while FGL2 can inhibit APC maturation and subsequently reduce T cell activation, the use of anti-FGL2 antibodies to antagonize FGL2 can activate T cells. Therefore, the development of FGL2 inhibitors may play an important role in the immunotherapy of gliomas (37).

### FGL2 is an effective molecule of Tregs

Regulatory cells (Tregs) are a subpopulation of T cells that controls autoimmune reactivity *in vivo*, first reported by Sakakuchi et al. in 1995 (66, 67). Tregs can be divided into nTregs and aTregs. nTregs are mainly composed of Foxp3+CD25+CD4+ Treg cells, which function through cell-to-cell contacts (68). In recent years, many scholars have conducted numerous studies on the immunological aspects of Tregs in tumors. These studies confirm the large amounts of Tregs infiltration in tumor tissues such as liver (69), ovarian (70), lung (71), breast (72), and gastrointestinal tumors (73), and their significant presence is often associated with poor tumor prognosis. Attempts have been made to explain the mechanisms by which Tregs suppress the immune response at various cellular and molecular levels (74). They include: 1, through cell-to-cell contact-dependent inhibition, Tregs cells are inhibited by CTLA-4 and TGF-β etc. directly binding to the corresponding receptors on target cells, reducing the responsiveness of target cells to IL-2 and thus inhibiting the proliferation of effector T cells. 2, relying on suppressive cytokines such as IL-4 and IL-10. 3, modulating the body’s immunity by interacting with antigen-presenting cells, suppressing effector T cells by downregulating the function of APC cells or competing for co-stimulatory molecules on APC cells (75–82).

FGL2, which has immunomodulatory activity, has been reported to be highly expressed in Treg by many studies (83–85). To verify the importance of FGL2 on Treg function, Itay Shalev et al. used an anti-FGL2 monoclonal antibody to antagonize Treg *in vitro*. The experimental results showed that the FGL2 antibody could completely inhibit Treg’s function. This result further supports the previous finding that FGL2 may inhibit T cell proliferation by suppressing the production of immune- activating cytokines IL-2 and IFN-γ and promoting the production of suppressive cytokines IL-4 and IL-1 (25, 26).

### FGL2 promote M2

Tumor-associated macrophages (TAM) are infiltrating macrophages in tumor tissues, which are mainly differentiated from monocytes (86, 87). Chemokines such as CSF1 and CCL2 secreted by tumor cells can recruit monocytes from peripheral circulating blood to the tumor microenvironment (TME), and then monocytes differentiate into macrophage (88, 89). A growing number of studies have shown that TAM has a series of tumor-promoting functions such as supporting tumor cell proliferation, invasion, and metastasis, and is highly correlated with poor prognosis of tumor patients (23, 90, 91). Based on phenotype and function, macrophages can be divided into two main types, M1 (pro-inflammatory, classically activated macrophages) and M2 (anti-inflammatory, alternatively activated macrophages (92–94). In addition, M1-type macrophages kill tumor cells and defend against pathogen invasion, while M2-type macrophages mainly play a role in promoting tumor growth, invasion and metastasis (95, 96).

In gliomas, TAMs include brain-resident microglia and bone marrow-derived macrophages, which account for 50% of GBM tumors (97).TAMs and glioma cells are important components in promoting glioma growth, and their interactions are critical for glioma proliferation, treatment resistance, and tumor recurrence (98). FGL2 may be a central effector molecule in this interaction. Yan et al. found differences in macrophage infiltration between FGL2hi and FGL2KO gliomas by flow cytometry analysis, demonstrating that FGL2 can increase the M2 macrophage population. Macrophage migration assays were performed by using FGL2hi and FGL2KO glioma cells conditioned medium (CM). It was found that CM from FGL2hi attracted more macrophage aggregation than that from FGL2KO. The migratory effect of macrophages was also significantly suppressed after blocking FGL2 or using antibody blockers of the FGL2 receptor FcγRIII (CD16) (23).These findings suggest that FGL2 acts as a potent chemokine that recruits macrophages into the tumor microenvironment (TME) of gliomas, and CD16 is the receptor that mediates this chemotactic effect. They also found that FGL2 secreted on glioma cells could bind to the receptor CD16 on TAMs and promote the Syk/PI3K/AKT/HIF1α signaling pathway, then induce the release of CXCL7, which in turn could act on glioma cells and facilitate their Stem-like transition (23). However, the relationship between FGL2, TAMs and gliomas cells has not been sufficiently studied, and more experiments are needed to verify the relationship in the future.

## Interaction of FGL2 with immune checkpoint molecules

**Figure 4:**
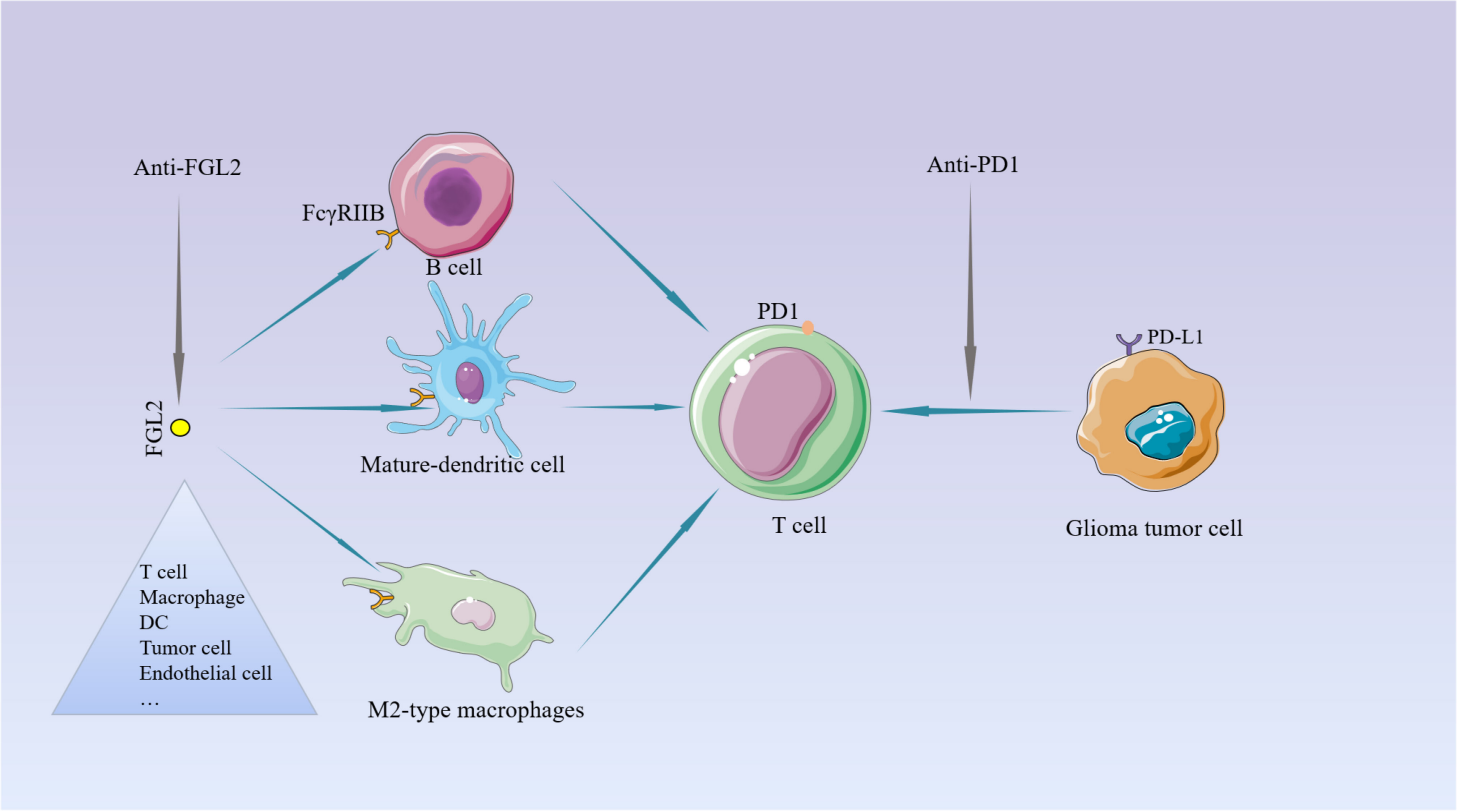
The potential for targeting FGL2. Unlike PD1 on T cells, FGL2 primarily targets the APC. PD-1 induces FGL2 expression by inhibiting IFN-γ and TNF-α.

The current treatment of the new GBM diagnosis is still focused on maximal resection and combined radiation and chemotherapy. Unfortunately, almost all treated patients relapse within a short period, and there are currently no effective treatments to prolong their survival for relapsed patients (99, 100). So, it is urgent to find a more efficient treatment. Over the past few years, immune checkpoint inhibition has gradually received more attention in treating tumors, providing a ray of hope for some tumors with limited traditional treatment options (101, 102). For example, PD-1 (Programmed cell death protein 1) checkpoint inhibitor can bind to PD-1 or its ligand PD-L1 and block PD-1 from blocking T cell (103).But the current effect of immune checkpoint inhibitors represented by PD-1 in the treatment of glioma is not very satisfactory (104, 105). Therefore, it is essential to find more effective immune targets. In a review of previous studies, we found that FGL2 acts more on antigen-presenting cells APCs upstream of T cells. These cells are present in the glioma microenvironment (37), and play a key role in immunosuppression by suppressing T cell proliferation. As mentioned repeatedly in the literature, targeting a single molecule or pathway is not sufficient to inhibit the malignant progression of gliomas, so if we can find a key point that can inhibit multiple immunosuppressive pathways or mechanisms, it is possible to achieve good therapeutic results. As a molecule with such a role, the development of inhibitors of FGL2 may help reverse immunosuppression in the tumor microenvironment and may play an essential role in targeting glioma-mediated immunosuppressive therapy. Combined with previous findings, PD-1 can elevate FGL2 expression by inhibiting IFN-r and TNF-a. It is not difficult to infer that simultaneous inhibition of IL-10 and PD-1 can increase T cell proliferation, which will be verified in the future (**Figure 4**). Ntv-a mice injected with RCA-PDGFB+FGL2 were treated with anti-FGL2 antibodies, and the control mice were treated with IgG antibodies. Statistical analysis of the survival of the mice revealed that the median asymptomatic survival of the anti-FGL2 antibody-treated mice was significantly longer than the control. The tumors treated with anti-FGL2 antibody were also found to have lower CD44 expression and higher Olig2 expression than the control group, indirectly demonstrating that high expression of FGL2 is associated with the mesenchymal subtype of glioma. Comparing the number of Treg in the two groups after 20 days of treatment, it was found that the anti-FGL2 antibody-treated mice showed fewer Treg along with a small number of arginase1+/Iba1+ macrophages. These suggest that anti-FGL2 antibody treatment can effectively prevent immunosuppression of the tumor microenvironment (106).

## FGL2 is involved in the formation of blood vessels in gliomas

Liu et al. established a stable knockdown of FGL2 in the HCCLM6 cell line and found that downregulation of FGL2 reduced tumor angiogenesis in HCCLM6 nude mouse xenograft. hFGL2 expression in HCC tumor cells promotes tumor growth and angiogenesis through activation of ERK and JNK pathways, and FGL2 protein secreted by tumor cells promotes angiogenesis and tumor growth through activation of the thrombin-dependent MAPK pathway. Extensive studies have shown that IL8 and VEGF are the most important activators of tumor-associated angiogenesis (107–109). Activation of multiple VEGF/VEGF receptor signaling pathways leads to endothelial cell survival, mitosis, migration, differentiation, vascular permeability, and endothelial progenitor cell mobilization (110, 111). IL-8 exerts its powerful angiogenic properties on endothelial cells through interaction with its receptors CXCR1 and CXCR2 (112, 113). In the nude mouse subcutaneous tumor model with stable downregulation of FGL2, FGL2 was found to be downregulated along with reduced expression of VEGF and IL-8 (28). At the same time, downregulation of FGL2 was found to affect angiogenesis in corneal microcapsule analysis in nude mice. However, FGL2 is also expressed in endothelial cells and macrophages, and FGL2 may also promote tumor angiogenesis by inducing value-added endothelial cells and recruiting inflammatory cells, this part needs to be further investigated. The correlation of FGL2 expression with enhanced phosphorylation of ERK and JNK strongly suggests that FGL2 plays an important role in tumor growth in HCC by regulating the activation of the MAPK pathway (36). FGL2 can lead to phosphorylation of ERK and p38 through thrombin production and subsequent activation of PAR1 and PAR3, or JNK through activation of PAR2, and these late cellular activities promote survival and proliferation, tumor growth and angiogenesis in hepatocellular carcinoma. These observations suggest that FGL2 prothrombinase, in conjunction with thrombin and tissue factor, may contribute to tumor hypercoagulability and possibly to angiogenesis and metastasis. In turn, FGL2 may serve as a novel target for the intervention of tumor development. Nevertheless, there are no relevant literature reports on whether FGL2 has an effect on tumor angiogenesis in glioma, and more experimental validation may be needed at a later stage.

## Conclusion

FGL2 is widely expressed in gliomas and also plays an essential immunosuppressive role, thereby promoting tumor immune escape and malignant progression. Since FGL2 can act on antigen-presenting cells and inhibit the anti-tumor activity of T cells, it may be a future direction to try to apply FGL2 targeting therapy to GBM treatment. In-depth understanding and elucidation of the transcriptional and signaling mechanisms of FGL2 in different cell types of gliomas are essential for developing new specific targeted drugs.

## Author contributions

SZ designed and led the project. XM wrote the manuscript. HZ, LC, XC, and KS provided valuable revisions to the manuscript. All authors contributed to this article and approved the submitted version.

## Funding

This work was supported by the National Natural Science Foundation of China, No. 82072805 and Hubei Natural Science Foundation, No. 2020CFB678.

## Conflict of interest

The authors declare that the research was conducted in the absence of any commercial or financial relationships that could be construed as a potential conflict of interest.

## Publisher’s note

All claims expressed in this article are solely those of the authors and do not necessarily represent those of their affiliated organizations, or those of the publisher, the editors and the reviewers. Any product that may be evaluated in this article, or claim that may be made by its manufacturer, is not guaranteed or endorsed by the publisher.

## References

[1] Goodenberger ML Jenkins RB. Genetics of adult glioma. Cancer Genet (2012) 205(12):613–21. doi: 10.1016/j.cancergen.2012.10.009 23238284

[2] Pop S Enciu AM Necula LG Tanase CP. Long non-coding RNAs in brain tumours: Focus on recent epigenetic findings in glioma. J Cell Mol Med (2018) 22(10):4597–610. doi: 10.1111/jcmm.13781 PMC6156469 30117678

[3] Louis DN Perry A Reifenberger G Deimling AV Figarella-Branger D Cavenee WK. The 2016 world health organization classification of tumors of the central nervous system: a summary. Acta Neuropathologica (2016) 131(6):803–20. doi: 10.1007/s00401-016-1545-1 27157931

[4] Schwartzbaum JA Fisher JL Aldape KD Wrensch M. Epidemiology and molecular pathology of glioma. Nat Clin Pract Neurol (2006) 2(9):494–503. doi: 10.1038/ncpneuro0289 16932614

[5] Molinaro AM Taylor JW Wiencke JK Wrensch MR. Genetic and molecular epidemiology of adult diffuse glioma. Nat Rev Neurol (2019) 15(7):405–17. doi: 10.1038/s41582-019-0220-2 PMC7286557 31227792

[6] Man J Yu X Huang H Zhou W Xiang C Huang H. Hypoxic induction of vasorin regulates Notch1 turnover to maintain glioma stem-like cells. Cell Stem Cell (2018) 22(1):104–118 e6. doi: 10.1016/j.stem.2017.10.005 29198941 PMC5756127

[7] Gusyatiner O Hegi ME. Glioma epigenetics: From subclassification to novel treatment options. Semin Cancer Biol (2018) 51:50–8. doi: 10.1016/j.semcancer.2017.11.010 29170066

[8] Cruceru ML Enciu AM Popa AC Albulescu R Neagu M Tanase CP. Signal transduction molecule patterns indicating potential glioblastoma therapy approaches. Onco Targets Ther (2013) 6:1737–49. doi: 10.2147/OTT.S52365 PMC3848931 24348050

[9] Tanase C Enciu AM Codrici E Popescu ID Dudau M Dobri AM. Fatty acids, CD36, thrombospondin-1, and CD47 in glioblastoma: Together and/or separately? Int J Mol Sci (2022) 23(2):604. doi: 10.3390/ijms23020604 35054787 PMC8776193

[10] Lamplugh Z Fan Y. Vascular microenvironment, tumor immunity and immunotherapy. Front Immunol (2021) 12:811485. doi: 10.3389/fimmu.2021.811485 34987525 PMC8720970

[11] Kennedy LB Salama AKS. A review of cancer immunotherapy toxicity. CA Cancer J Clin (2020) 70(2):86–104. doi: 10.3322/caac.21596 31944278

[12] O'Donnell JS Teng MWL Smyth MJ. Cancer immunoediting and resistance to T cell-based immunotherapy. Nat Rev Clin Oncol (2019) 16(3):151–67. doi: 10.1038/s41571-018-0142-8 30523282

[13] Sanmamed MF Chen L. A paradigm shift in cancer immunotherapy: From enhancement to normalization. Cell (2018) 175(2):313–26. doi: 10.1016/j.cell.2018.09.035 PMC6538253 30290139

[14] Ghouzlani A Kandoussi S Tall M Reddy KP Rafii S Badou A. Immune checkpoint inhibitors in human glioma microenvironment. Front Immunol (2021) 12:679425. doi: 10.3389/fimmu.2021.679425 34305910 PMC8301219

[15] Kurz SC Cabrera LP Hastie D Huang R Unadkat P Rinne M. PD-1 inhibition has only limited clinical benefit in patients with recurrent high-grade glioma. Neurology (2018) 91(14):e1355–9. doi: 10.1212/WNL.0000000000006283 30171077

[16] Gieryng A Pszczolkowska D Walentynowicz KA Rajan WD Kaminska B. Immune microenvironment of gliomas. Lab Invest (2017) 97(5):498–518. doi: 10.1038/labinvest.2017.19 28287634

[17] Fecci PE Mitchell DA Whitesides JF Xie W Friedman AH Archer GE. Increased regulatory T-cell fraction amidst a diminished CD4 compartment explains cellular immune defects in patients with malignant glioma. Cancer Res (2006) 66(6):3294–302. doi: 10.1158/0008-5472.CAN-05-3773 16540683

[18] Lohr J Ratliff T Huppertz A Ge Y Dictus C Ahmadi R. Effector T-cell infiltration positively impacts survival of glioblastoma patients and is impaired by tumor-derived TGF-beta. Clin Cancer Res (2011) 17(13):4296–308. doi: 10.1158/1078-0432.CCR-10-2557 21478334

[19] Lim M Xia Y Bettegowda C Weller M. Current state of immunotherapy for glioblastoma. Nat Rev Clin Oncol (2018) 15(7):422–42. doi: 10.1038/s41571-018-0003-5 29643471

[20] Maire CL Mohme M Bockmayr M Fita KD Riecken K Bornigen D. Glioma escape signature and clonal development under immune pressure. J Clin Invest (2020) 130(10):5257–71. doi: 10.1172/JCI138760 PMC7524465 32603315

[21] Rosenberg SA Restifo NP. Adoptive cell transfer as personalized immunotherapy for human cancer. Science (2015) 348(6230):62–8. doi: 10.1126/science.aaa4967 PMC6295668 25838374

[22] Yan J Zhao Q Gabrusiewicz K Kong LY Xia X Wang J. FGL2 promotes tumor progression in the CNS by suppressing CD103(+) dendritic cell differentiation. Nat Commun (2019) 10(1):448. doi: 10.1038/s41467-018-08271-x 30683885 PMC6347641

[23] Yan J Zhao Q Wang J Tian X Wang J Xia X. FGL2-wired macrophages secrete CXCL7 to regulate the stem-like functionality of glioma cells. Cancer Lett (2021) 506:83–94. doi: 10.1016/j.canlet.2021.02.021 33676940 PMC8009861

[24] Liu H Yang PS Zhu T Manuel J Zhang J He W. Characterization of fibrinogen-like protein 2 (FGL2): monomeric FGL2 has enhanced immunosuppressive activity in comparison to oligomeric FGL2. Int J Biochem Cell Biol (2013) 45(2):408–18. doi: 10.1016/j.biocel.2012.10.014 23127799

[25] Chan CW Kay LS Khadaroo RG Chan MW Lakatoo S Young KJ. Soluble fibrinogen-like protein 2/fibroleukin exhibits immunosuppressive properties: suppressing T cell proliferation and inhibiting maturation of bone marrow-derived dendritic cells. J Immunol (2003) 170(8):4036–44. doi: 10.4049/jimmunol.170.8.4036 12682232

[26] Shalev I Liu H Koscik C Bartczak A Javadi M Wong KM. Targeted deletion of fgl2 leads to impaired regulatory T cell activity and development of autoimmune glomerulonephritis. J Immunol (2008) 180(1):249–60. doi: 10.4049/jimmunol.180.1.249 18097026

[27] Hu J Yan J Rao G Latha K Overwijk WW Heimberger AB. The duality of Fgl2 - secreted immune checkpoint regulator versus membrane-associated procoagulant: Therapeutic potential and implications. Int Rev Immunol (2016) 35(4):325–39. doi: 10.3109/08830185.2014.956360 PMC5257246 25259408

[28] Liu Y Xu S Xiao F Xiong Y Wang X Gao S. The FGL2/fibroleukin prothrombinase is involved in alveolar macrophage activation in COPD through the MAPK pathway. Biochem Biophys Res Commun (2010) 396(2):555–61. doi: 10.1016/j.bbrc.2010.04.145 20438701

[29] Dong X Ye X Chen X Chen T Xie S Li Q. Intestinal and peripheral fibrinogen-like protein 2 expression in inflammatory bowel disease. Dig Dis Sci (2014) 59(4):769–77. doi: 10.1007/s10620-013-2962-9 PMC7087870 24287641

[30] Yan J Kong LY Hu J Gabrusiewicz K Dibra D Xia X. FGL2 as a multimodality regulator of tumor-mediated immune suppression and therapeutic target in gliomas. J Natl Cancer Inst (2015) 107(8):djv137. doi: 10.1093/jnci/djv137 25971300 PMC4554195

[31] Song Z Wang Y Du Y Zhang Z Yuan Y. Identification of integrative molecular and clinical profiles of fibrinogen-like protein 2 in gliomas using 1323 samples. Int Immunopharmacol (2020) 88:106894. doi: 10.1016/j.intimp.2020.106894 32858440

[32] Yu J Li J Shen J Du F Wu X Li M. The role of fibrinogen-like proteins in cancer. Int J Biol Sci (2021) 17(4):1079–87. doi: 10.7150/ijbs.56748 PMC8040309 33867830

[33] Zhu Y Zhang L Zha H Yang F Hu C Chen L. Stroma-derived fibrinogen-like protein 2 activates cancer-associated fibroblasts to promote tumor growth in lung cancer. Int J Biol Sci (2017) 13(6):804–14. doi: 10.7150/ijbs.19398 PMC5485635 28656005

[34] Liu X Chu Y Wang D Weng Y Jia Z. MAPK-mediated upregulation of fibrinogen-like protein 2 promotes proliferation, migration, and invasion of colorectal cancer cells. Cell Biol Int (2019) 43(13):1483–91. doi: 10.1002/cbin.11198 31286589

[35] Qin WZ Li QL Chen WF Xu MD Zhang YQ Zhong YS. Overexpression of fibrinogen-like protein 2 induces epithelial-to-mesenchymal transition and promotes tumor progression in colorectal carcinoma. Med Oncol (2014) 31(9):181. doi: 10.1007/s12032-014-0181-7 25129313 PMC7090555

[36] Liu Y Xu L Zeng Q Wang J Wang M Xi D. Downregulation of FGL2/prothrombinase delays HCCLM6 xenograft tumour growth and decreases tumour angiogenesis. Liver Int (2012) 32(10):1585–95. doi: 10.1111/j.1478-3231.2012.02865.x 22925132

[37] Patel R Traylor JI Latha K Heimberger AB Li S Rao G. Fibrinogen-like protein 2: a potential molecular target for glioblastoma treatment. Expert Opin Ther Targets (2019) 23(8):647–9. doi: 10.1080/14728222.2019.1628220 PMC6635074 31167575

[38] van de Laar L Coffer PJ Woltman AM. Regulation of dendritic cell development by GM-CSF: molecular control and implications for immune homeostasis and therapy. Blood (2012) 119(15):3383–93. doi: 10.1182/blood-2011-11-370130 22323450

[39] Liu BQ Bao ZY Zhu JY Liu H. Fibrinogen-like protein 2 promotes the accumulation of myeloid-derived suppressor cells in the hepatocellular carcinoma tumor microenvironment. Oncol Lett (2021) 21(1):47. doi: 10.3892/ol.2020.12308 33281958 PMC7709556

[40] Wang M Liu J Xi D Luo X Ning Q. Adenovirus-mediated artificial microRNA against human fibrinogen like protein 2 inhibits hepatocellular carcinoma growth. J Gene Med (2016) 18(7):102–11. doi: 10.1002/jgm.2883 27163335

[41] Lee YS Radford KJ. The role of dendritic cells in cancer. Int Rev Cell Mol Biol (2019) 348:123–78. doi: 10.1016/bs.ircmb.2019.07.006 31810552

[42] Bol KF Schreibelt G Rabold K Wculek SK Schwarze JK Dzionek A. The clinical application of cancer immunotherapy based on naturally circulating dendritic cells. J Immunother Cancer (2019) 7(1):109. doi: 10.1186/s40425-019-0580-6 30999964 PMC6471787

[43] Gardner A Ruffell B. Dendritic cells and cancer immunity. Trends Immunol (2016) 37(12):855–65. doi: 10.1016/j.it.2016.09.006 PMC5135568 27793569

[44] Pitt JM André F Amigorena S Soria JC Eggermont A Kroeme RG. Dendritic cell-derived exosomes for cancer therapy. J Clin Invest (2016) 126(4):1224–32. doi: 10.1172/JCI81137 PMC4811123 27035813

[45] Collin M Bigley V. Human dendritic cell subsets: an update. Immunology (2018) 154(1):3–20. doi: 10.1111/imm.12888 29313948 PMC5904714

[46] Bol KF Schreibelt G Gerritsen WR de Vries IJ Figdor CG. Dendritic cell-based immunotherapy: State of the art and beyond. Clin Cancer Res (2016) 22(8):1897–906. doi: 10.1158/1078-0432.CCR-15-1399 27084743

[47] Del Balzo D Capmany A Cebrian I Damiani MT. Chlamydia trachomatis infection impairs MHC-I intracellular trafficking and antigen cross-presentation by dendritic cells. Front Immunol (2021) 12:662096. doi: 10.3389/fimmu.2021.662096 33936099 PMC8082151

[48] Horrevorts SK Duinkerken S Bloem K Secades P Kalay H Musters RJ. Toll-like receptor 4 triggering promotes cytosolic routing of DC-SIGN-Targeted antigens for presentation on MHC class I. Front Immunol (2018) 9:1231. doi: 10.3389/fimmu.2018.01231 29963041 PMC6010527

[49] van Niel G Wubbolts R Stoorvogel W. Endosomal sorting of MHC class II determines antigen presentation by dendritic cells. Curr Opin Cell Biol (2008) 20(4):437–44. doi: 10.1016/j.ceb.2008.05.011 18582577

[50] Palucka K Banchereau J. Cancer immunotherapy via dendritic cells. Nat Rev Cancer (2012) 12(4):265–77. doi: 10.1038/nrc3258 PMC3433802 22437871

[51] Radford KJ Tullett KM Lahoud MH. Dendritic cells and cancer immunotherapy. Curr Opin Immunol (2014) 27:26–32. doi: 10.1016/j.coi.2014.01.005 24513968

[52] Wculek SK Cueto FJ Mujal AM Melero I Krummel MF Sancho D. Dendritic cells in cancer immunology and immunotherapy. Nat Rev Immunol (2020) 20(1):7–24. doi: 10.1038/s41577-019-0210-z 31467405

[53] Roberts EW Broz ML Binnewies M Headley MB Nelson AE Wolf DM. Critical role for CD103(+)/CD141(+) dendritic cells bearing CCR7 for tumor antigen trafficking and priming of T cell immunity in melanoma. Cancer Cell (2016) 30(2):324–36. doi: 10.1016/j.ccell.2016.06.003 PMC5374862 27424807

[54] Galluzzi L Humeau J Buqué A Zitvogel L Kroemer G. Immunostimulation with chemotherapy in the era of immune checkpoint inhibitors. Nat Rev Clin Oncol (2020) 17(12):725–41. doi: 10.1038/s41571-020-0413-z 32760014

[55] Perng P Lim M. Immunosuppressive mechanisms of malignant gliomas: Parallels at non-CNS sites. Front Oncol (2015) 5:153. doi: 10.3389/fonc.2015.00153 26217588 PMC4492080

[56] Belz GT Nutt SL. Transcriptional programming of the dendritic cell network. Nat Rev Immunol (2012) 12(2):101–13. doi: 10.1038/nri3149 22273772

[57] Zhan Y Xu Y Lew AM. The regulation of the development and function of dendritic cell subsets by GM-CSF: more than a hematopoietic growth factor. Mol Immunol (2012) 52(1):30–7. doi: 10.1016/j.molimm.2012.04.009 22580403

[58] Kishton RJ Sukumar M Restifo NP. Metabolic regulation of T cell longevity and function in tumor immunotherapy. Cell Metab (2017) 26(1):94–109. doi: 10.1016/j.cmet.2017.06.016 28683298 PMC5543711

[59] Schwartz RH. T Cell anergy. Annu Rev Immunol (2003) 21:305–34. doi: 10.1146/annurev.immunol.21.120601.141110 12471050

[60] Choi Y Shi Y Haymaker CL Naing A Ciliberto G Hajjar J. T-Cell agonists in cancer immunotherapy. J Immunother Cancer (2020) 8(2):e000966. doi: 10.1136/jitc-2020-000966 33020242 PMC7537335

[61] van der Leun AM Thommen DS Schumacher TN. CD8(+) T cell states in human cancer: insights from single-cell analysis. Nat Rev Cancer (2020) 20(4):218–32. doi: 10.1038/s41568-019-0235-4 PMC7115982 32024970

[62] Reina-Campos M Scharping NE Goldrath AW. CD8(+) T cell metabolism in infection and cancer. Nat Rev Immunol (2021) 21(11):718–38. doi: 10.1038/s41577-021-00537-8 PMC8806153 33981085

[63] Han J Khatwani N Searles TG Turk MJ Angeles CV. Memory CD8(+) T cell responses to cancer. Semin Immunol (2020) 49:101435. doi: 10.1016/j.smim.2020.101435 33272898 PMC7738415

[64] Borst J Ahrends T Babala N Melief CJM Kastenmuller W. CD4(+) T cell help in cancer immunology and immunotherapy. Nat Rev Immunol (2018) 18(10):635–47. doi: 10.1038/s41577-018-0044-0 30057419

[65] Zander R Schauder D Xin G Nguyen C Wu X Zajac A. CD4(+) T cell help is required for the formation of a cytolytic CD8(+) T cell subset that protects against chronic infection and cancer. Immunity (2019) 51(6):1028–1042 e4. doi: 10.1016/j.immuni.2019.10.009 31810883 PMC6929322

[66] Ohue Y Nishikawa H. Regulatory T (Treg) cells in cancer: Can treg cells be a new therapeutic target? Cancer Sci (2019) 110(7):2080–9. doi: 10.1111/cas.14069 PMC6609813 31102428

[67] Tanaka A Sakaguchi S. Targeting treg cells in cancer immunotherapy. Eur J Immunol (2019) 49(8):1140–6. doi: 10.1002/eji.201847659 31257581

[68] Tanaka A Sakaguchi S. Regulatory T cells in cancer immunotherapy. Cell Res (2017) 27(1):109–18. doi: 10.1038/cr.2016.151 PMC5223231 27995907

[69] Aghabi YO Yasin A Kennedy JI Davies SP Butler AE Stamataki Z. Targeting enclysis in liver autoimmunity, transplantation, viral infection and cancer. Front Immunol (2021) 12:662134. doi: 10.3389/fimmu.2021.662134 33953725 PMC8089374

[70] Zhou J Li X Wu X Zhang T Zhu Q Wang X. Exosomes released from tumor-associated macrophages transfer miRNAs that induce a Treg/Th17 cell imbalance in epithelial ovarian cancer. Cancer Immunol Res (2018) 6(12):1578–92. doi: 10.1158/2326-6066.CIR-17-0479 30396909

[71] Xie M Wei J Xu J. Inducers, attractors and modulators of CD4(+) treg cells in non-Small-Cell lung cancer. Front Immunol (2020) 11:676. doi: 10.3389/fimmu.2020.00676 32425930 PMC7212357

[72] Bai F Zhang P Fu Y Chen H Zhang M Huang Q. Targeting ANXA1 abrogates treg-mediated immune suppression in triple-negative breast cancer. J Immunother Cancer (2020) 8(1):e000169. doi: 10.1136/jitc-2019-000169 32300050 PMC7204868

[73] Ji L Qian W Gui L Ji Z Yin P Lin GN. Blockade of beta-Catenin-Induced CCL28 suppresses gastric cancer progression via inhibition of treg cell infiltration. Cancer Res (2020) 80(10):2004–16. doi: 10.1158/0008-5472.CAN-19-3074 32156780

[74] Togashi Y Shitara K Nishikawa H. Regulatory T cells in cancer immunosuppression - implications for anticancer therapy. Nat Rev Clin Oncol (2019) 16(6):356–71. doi: 10.1038/s41571-019-0175-7 30705439

[75] Barbi J Pardoll D Pan F. Treg functional stability and its responsiveness to the microenvironment. Immunol Rev (2014) 259(1):115–39. doi: 10.1111/imr.12172 PMC3996455 24712463

[76] Josefowicz SZ Lu LF Rudensky AY. Regulatory T cells: mechanisms of differentiation and function. Annu Rev Immunol (2012) 30:531–64. doi: 10.1146/annurev.immunol.25.022106.141623 PMC6066374 22224781

[77] Benson MJ Pino-Lagos K Rosemblatt M Noelle RJ. All-trans retinoic acid mediates enhanced T reg cell growth, differentiation, and gut homing in the face of high levels of co-stimulation. J Exp Med (2007) 204(8):1765–74. doi: 10.1084/jem.20070719 PMC2118687 17620363

[78] Xu L Kitani A Fuss I Strober W. Cutting edge: regulatory T cells induce CD4+CD25-Foxp3- T cells or are self-induced to become Th17 cells in the absence of exogenous TGF-beta. J Immunol (2007) 178(11):6725–9. doi: 10.4049/jimmunol.178.11.6725 17513718

[79] Rubtsov YP Rasmussen JP Chi EY Fontenot J Castelli L Ye X. Regulatory T cell-derived interleukin-10 limits inflammation at environmental interfaces. Immunity (2008) 28(4):546–58. doi: 10.1016/j.immuni.2008.02.017 18387831

[80] Zhang L Jarvis LB Baek HJ Gaston JS. Regulatory IL4+CD8+ T cells in patients with ankylosing spondylitis and healthy controls. Ann Rheum Dis (2009) 68(8):1345–51. doi: 10.1136/ard.2008.088120 18647857

[81] Tekguc M Wing JB Osaki M Long J Sakaguchi S. Treg-expressed CTLA-4 depletes CD80/CD86 by trogocytosis, releasing free PD-L1 on antigen-presenting cells. Proc Natl Acad Sci U.S.A. (2021) 118(30):e2023739118. doi: 10.1073/pnas.2023739118 34301886 PMC8325248

[82] Dai E Zhu Z Wahed S Qu Z Storkus WJ Guo ZS. Epigenetic modulation of antitumor immunity for improved cancer immunotherapy. Mol Cancer (2021) 20(1):171. doi: 10.1186/s12943-021-01464-x 34930302 PMC8691037

[83] Shalev I Wong KM Foerster K Zhu Y Chan C Maknojia A. The novel CD4+CD25+ regulatory T cell effector molecule fibrinogen-like protein 2 contributes to the outcome of murine fulminant viral hepatitis. Hepatology (2009) 49(2):387–97. doi: 10.1002/hep.22684 19085958

[84] Foerster K Helmy A Zhu Y Khattar R Adeyi OA Wong KM. The novel immunoregulatory molecule FGL2: a potential biomarker for severity of chronic hepatitis c virus infection. J Hepatol (2010) 53(4):608–15. doi: 10.1016/j.jhep.2010.04.020 20615566

[85] Zhao Z Yang C Wang L Li L Zhao T Hu L. The regulatory T cell effector soluble fibrinogen-like protein 2 induces tubular epithelial cell apoptosis in renal transplantation. Exp Biol Med (Maywood) (2014) 239(2):193–201. doi: 10.1177/1535370213514921 24414480

[86] Vitale I Manic G Coussens LM Kroemer G Galluzzi L. Macrophages and metabolism in the tumor microenvironment. Cell Metab (2019) 30(1):36–50. doi: 10.1016/j.cmet.2019.06.001 31269428

[87] Schulz M Sevenich L. TAMs in brain metastasis: Molecular signatures in mouse and man. Front Immunol (2021) 12:716504. doi: 10.3389/fimmu.2021.716504 34539650 PMC8447936

[88] Mantovani A Marchesi F Malesci A Laghi L Allavena P. Tumour-associated macrophages as treatment targets in oncology. Nat Rev Clin Oncol (2017) 14(7):399–416. doi: 10.1038/nrclinonc.2016.217 28117416 PMC5480600

[89] Yang Q Guo N Zhou Y Chen J Wei Q Han M. The role of tumor-associated macrophages (TAMs) in tumor progression and relevant advance in targeted therapy. Acta Pharm Sin B (2020) 10(11):2156–70. doi: 10.1016/j.apsb.2020.04.004 PMC7714989 33304783

[90] Qiu SQ Waaijer SJH Zwager MC de Vries EGE van der Vegt B Schroder CP. Tumor-associated macrophages in breast cancer: Innocent bystander or important player? Cancer Treat Rev (2018) 70:178–89. doi: 10.1016/j.ctrv.2018.08.010 30227299

[91] Wenes M Shang M Di Matteo M Goveia J Martín-Pérez R Serneels J. Macrophage metabolism controls tumor blood vessel morphogenesis and metastasis. Cell Metab (2016) 24(5):701–15. doi: 10.1016/j.cmet.2016.09.008 27773694

[92] Pan Y Yu Y Wang X Zhang T. Tumor-associated macrophages in tumor immunity. Front Immunol (2020) 11:583084. doi: 10.3389/fimmu.2020.583084 33365025 PMC7751482

[93] Xia Y Rao L Yao H Wang Z Ning P Chen X. Engineering macrophages for cancer immunotherapy and drug delivery. Adv Mater (2020) 32(40):e2002054. doi: 10.1002/adma.202002054 32856350

[94] Wu K Lin K Li X Yuan X Xu P Ni P. Redefining tumor-associated macrophage subpopulations and functions in the tumor microenvironment. Front Immunol (2020) 11:1731. doi: 10.3389/fimmu.2020.01731 32849616 PMC7417513

[95] Chen Y Song Y Du W Gong L Chang H Zou Z. Tumor-associated macrophages: an accomplice in solid tumor progression. J BioMed Sci (2019) 26(1):78. doi: 10.1186/s12929-019-0568-z 31629410 PMC6800990

[96] Lin Y Xu J Lan H. Tumor-associated macrophages in tumor metastasis: biological roles and clinical therapeutic applications. J Hematol Oncol (2019) 12(1):76. doi: 10.1186/s13045-019-0760-3 31300030 PMC6626377

[97] Widodo SS Dinevska M Furst LM Stylli SS Mantamadiotis T. IL-10 in glioma. Br J Cancer (2021) 125(11):1466–76. doi: 10.1038/s41416-021-01515-6 PMC8609023 34349251

[98] Zhou W Ke SQ Huang Z Flavahan W Fang X Paul J. Periostin secreted by glioblastoma stem cells recruits M2 tumour-associated macrophages and promotes malignant growth. Nat Cell Biol (2015) 17(2):170–82. doi: 10.1038/ncb3090 PMC4312504 25580734

[99] Tawbi HA Forsyth PA Algazi A Hamid O Hodi FS Moschos SJ. Combined nivolumab and ipilimumab in melanoma metastatic to the brain. N Engl J Med (2018) 379(8):722–30. doi: 10.1056/NEJMoa1805453 PMC8011001 30134131

[100] Weller M van den Bent M Hopkins K Tonn JC Stupp R Falini A. EANO guideline for the diagnosis and treatment of anaplastic gliomas and glioblastoma. Lancet Oncol (2014) 15(9):e395–403. doi: 10.1016/S1470-2045(14)70011-7 25079102

[101] Hodi FS O'Day SJ McDermott DF Weber RW Sosman JA Haanen JB. Improved survival with ipilimumab in patients with metastatic melanoma. N Engl J Med (2010) 363(8):711–23. doi: 10.1056/NEJMoa1003466 PMC3549297 20525992

[102] O'Day SJ Maio M Chiarion-Sileni V Gajewski TF Pehamberger H Bondarenko IN. Efficacy and safety of ipilimumab monotherapy in patients with pretreated advanced melanoma: a multicenter single-arm phase II study. Ann Oncol (2010) 21(8):1712–7. doi: 10.1093/annonc/mdq013 20147741

[103] Preusser M Lim M Hafler DA Reardon DA Sampson JH. Prospects of immune checkpoint modulators in the treatment of glioblastoma. Nat Rev Neurol (2015) 11(9):504–14. doi: 10.1038/nrneurol.2015.139 PMC4782584 26260659

[104] Zhao J Chen AX Gartrell RD Silverman AM Aparicio L Chu T. Immune and genomic correlates of response to anti-PD-1 immunotherapy in glioblastoma. Nat Med (2019) 25(3):462–9. doi: 10.1038/s41591-019-0349-y PMC6810613 30742119

[105] Lu Y Ng AHC Chow FE Everson RG Helmink BA Tetzlaff MT. Resolution of tissue signatures of therapy response in patients with recurrent GBM treated with neoadjuvant anti-PD1. Nat Commun (2021) 12(1):4031. doi: 10.1038/s41467-021-24293-4 34188042 PMC8241935

[106] Latha K Yan J Yang Y Gressot LV Kong LY Manyam G. The role of fibrinogen-like protein 2 on immunosuppression and malignant progression in glioma. J Natl Cancer Inst (2019) 111(3):292–300. doi: 10.1093/jnci/djy107 29947810 PMC6410946

[107] Martin D Galisteo R Gutkind JS. CXCL8/IL8 stimulates vascular endothelial growth factor (VEGF) expression and the autocrine activation of VEGFR2 in endothelial cells by activating NFkappaB through the CBM (Carma3/Bcl10/Malt1) complex. J Biol Chem (2009) 284(10):6038–42. doi: 10.1074/jbc.C800207200 PMC2649103 19112107

[108] Funakoshi T Lee CH Hsieh JJ. A systematic review of predictive and prognostic biomarkers for VEGF-targeted therapy in renal cell carcinoma. Cancer Treat Rev (2014) 40(4):533–47. doi: 10.1016/j.ctrv.2013.11.008 24398141

[109] Urbantat RM Blank A Kremenetskaia I Vajkoczy P Acker G Brandenburg S. The CXCL2/IL8/CXCR2 pathway is relevant for brain tumor malignancy and endothelial cell function. Int J Mol Sci (2021) 22(5):2634. doi: 10.3390/ijms22052634 33807899 PMC7961945

[110] Apte RS Chen DS Ferrara N. VEGF in signaling and disease: Beyond discovery and development. Cell (2019) 176(6):1248–64. doi: 10.1016/j.cell.2019.01.021 PMC6410740 30849371

[111] Goel HL Mercurio AM. VEGF targets the tumour cell. Nat Rev Cancer (2013) 13(12):871–82. doi: 10.1038/nrc3627 PMC4011842 24263190

[112] Waugh DJ Wilson C. The interleukin-8 pathway in cancer. Clin Cancer Res (2008) 14(21):6735–41. doi: 10.1158/1078-0432.CCR-07-4843 18980965

[113] Jin L Tao H Karachi A Long Y Hou AY Na M. CXCR1- or CXCR2-modified CAR T cells co-opt IL-8 for maximal antitumor efficacy in solid tumors. Nat Commun (2019) 10(1):4016. doi: 10.1038/s41467-019-11869-4 31488817 PMC6728370

